# Effect of microwave treatment on the physicochemical properties of potato starch granules

**DOI:** 10.1186/1752-153X-7-113

**Published:** 2013-07-08

**Authors:** Yanli Xie, Mingxia Yan, Shasha Yuan, Shumin Sun, Quangong Huo

**Affiliations:** 1 School of Food Science and Technology, Henan University of Technology, 1# Lianhua Street, High-tech Industrial Development Zone, Zhengzhou 450001, Henan, China

**Keywords:** Microwave treatment, Potato starch, Physicochemical properties

## Abstract

**Background:**

The degree of polymerization of amylose starch in potato was so large that the gel was hardness after gelatinization. Therefore, it is one of the most important ways that the microwave treatment was used to change the physicochemical properties of starch gel to make it suitable for the preparation of instant food.

**Results:**

The effect of microwave treatment on the physicochemical properties including morphology, crystalline structure, molecular weight distribution and rheological properties of potato starch granules was evaluated by treating time of varying duration (0, 5, 10, 15, 20 s) at 2450 MHz and 750 W. Scanning electron micrographs (SEM) of potato starch granules showed flaws or fractures on the surface after 5 to 10s of microwaving and collapse after 15 to 20 s. Polarized light microscopy (PLM) indicated that microwave treating damaged the crystalline structure of potato starch, such that the birefringence of starch granules gradually decreased after 5 to 10s and even disappeared after microwaving from 15 to 20 s. The molecular weight (Mw) values of potato starch and the proportion of large M_W_ fraction were considerably reduced with increasing the microwave treating time from 0 to 20s. The molecular weight slowly decreased over 5 ~ 15 s microwave treating but decreased abruptly at the time of 20s microwave treating. The apparent viscosity decreased as shear rate increased and presented shear-thinning behavior. The magnitudes of the storage modulus (*G*’) and loss modulus (*G”*) obtained at each shear rate increased with duration of microwave treating from 0 to 15 s but decreased from 15 to 20 s.

**Conclusions:**

These results demonstrated that the morphology and crystalline structure was damaged by microwave treatment. The high molecular weight of potato starch above 2 × 10^8^ Da was so sensitive to the vibrational motion of the polar molecules due to the application microwave energy and broke easily for longer dextran chains. The fracture of starch granules, molecular chains leached from the starch granules and degradation of dextran chains contributing to the development of rheological properties.

## Background

Microwave technologies have found widespread applications in various food processing operations [1]. As one of the most widely used ingredients, starch contributes to the structure, texture and consistency of processed foods. The effects of microwaving on the texture and nutritional properties of starch have also been studied [2]. The gels formed by microwaving differ significantly from those heated by conduction in terms of enzyme susceptibility, firmness and amylopectin recrystallization [3]. The most pronounced change induced by microwaving was the converting of potato starch crystal structure from Type B to Type A [4]. Microwaving has also been used to produce instant noodles from partially pre-gelatinized wheat flour dough [5,6]. The viscosity of both waxy and non-waxy starches showed significant changes after microwaving [7]. Microwave processing also raises the pasting temperature of lentil starch [8] and significantly lowers the firmness of the non-waxy rice starch gels in comparison to conventional heating. The differences in starch gels between heating by using microwave energy and conduction method can be attributed to the mechanism of heating [3]. Meanwhile, microwave thawing had a weaker effect than water bath on the viscoelasticity, microstructure and thermo graphic characteristics of starch-based sauces [9]. The susceptibility of different starches to microwave irradiation depended not only on their crystalline structure, but also on amylose content [10,11]. The degree of gelatinization of corn starch dispersions was significantly lower and slower than for wheat and rice starch after 15 to 25 s of microwave heating. Beyond 25 to 30 s of heating, differences in the gelatinization rates of wheat, corn and rice starch dispersions became non-significant, as measured by differential scanning calorimetry [12]. Potato starch formed networks from 0.3 to 11.0 nm in height and atomic force microscopy (AFM) detected that corn starches did not show any networks under microwave radiation. Heating mode influences potato starch far more than corn starch. China is the world’s largest producer of potatoes with annual outputs of ca. 70 million tons, some of which go into the production of starch and starchy foods [13] led by potato vermicelli, a Chinese staple, but the high firmness of retrograded potato starch demands long cooking time. This study aimed to evaluate the effect of microwave radiation on the physicochemical properties of potato starch. Our results are useful to select a processing technology for the development of new instant foods and other industrial applications.

## Results and discussion

### Starch granules morphology

**Figure 1:**
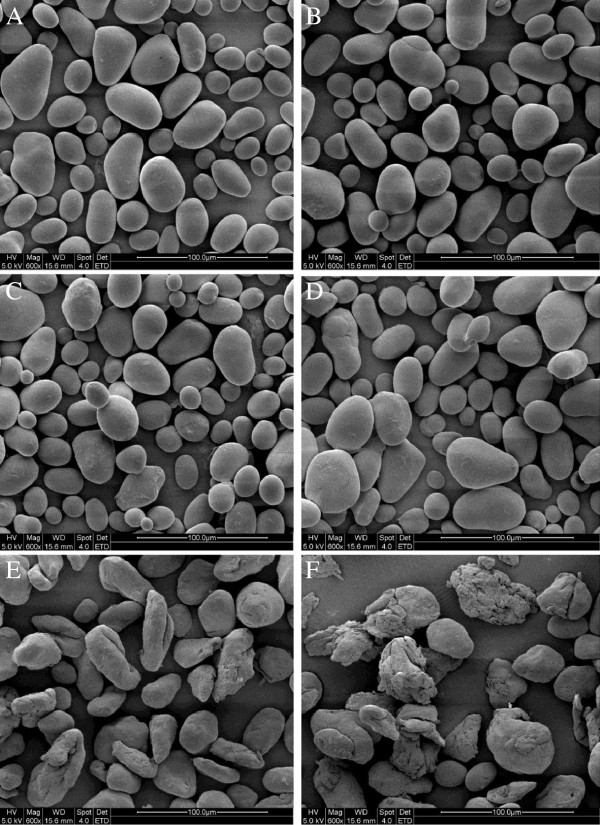
**SEM micrographs of native and microwave-treated starches. A**, **B**-Native starch: **C**, **D**, **E**, **F**-microwave treated for 5, 10, 15 and 20 s respectively.

SEM micrographs of the native starch and microwave-treated starches granules are presented in Figure 1. SEM showed that microwave treating changed the structure of starch granules [13]. Native starch granules showed a clear, regular elliptical shape with smooth surfaces. After 5 s of microwave treating and the final temperatures of 40°C, rare flaws or fractures were detected on the surface of some starch granules. After 10 s of microwaving and the final temperatures of 55°C, most starch granules showed noticeable flaws or fractures but no compromise to granule integrity. Further microwave treating for 15 s and the final temperatures of 80°C, it caused granule surfaces to appear rough and crimpy [1,8], with loss of integrity due to deformation and rupture in most cases [3]. Treating for 20 s and the final temperatures of 95°C caused heavy deformation, fracturing, and collapse of most starch granules. It has been reported that the treating method clearly affects the morphology of potato starch. Conventional treating causes surface gelatinization in the granules while microwave treating disrupts them totally because microwave energy affects the water molecules present in the crystalline regions of starch granules and enhances rupture [14].

### Crystalline structure of microwave-treated starch

**Figure 2:**
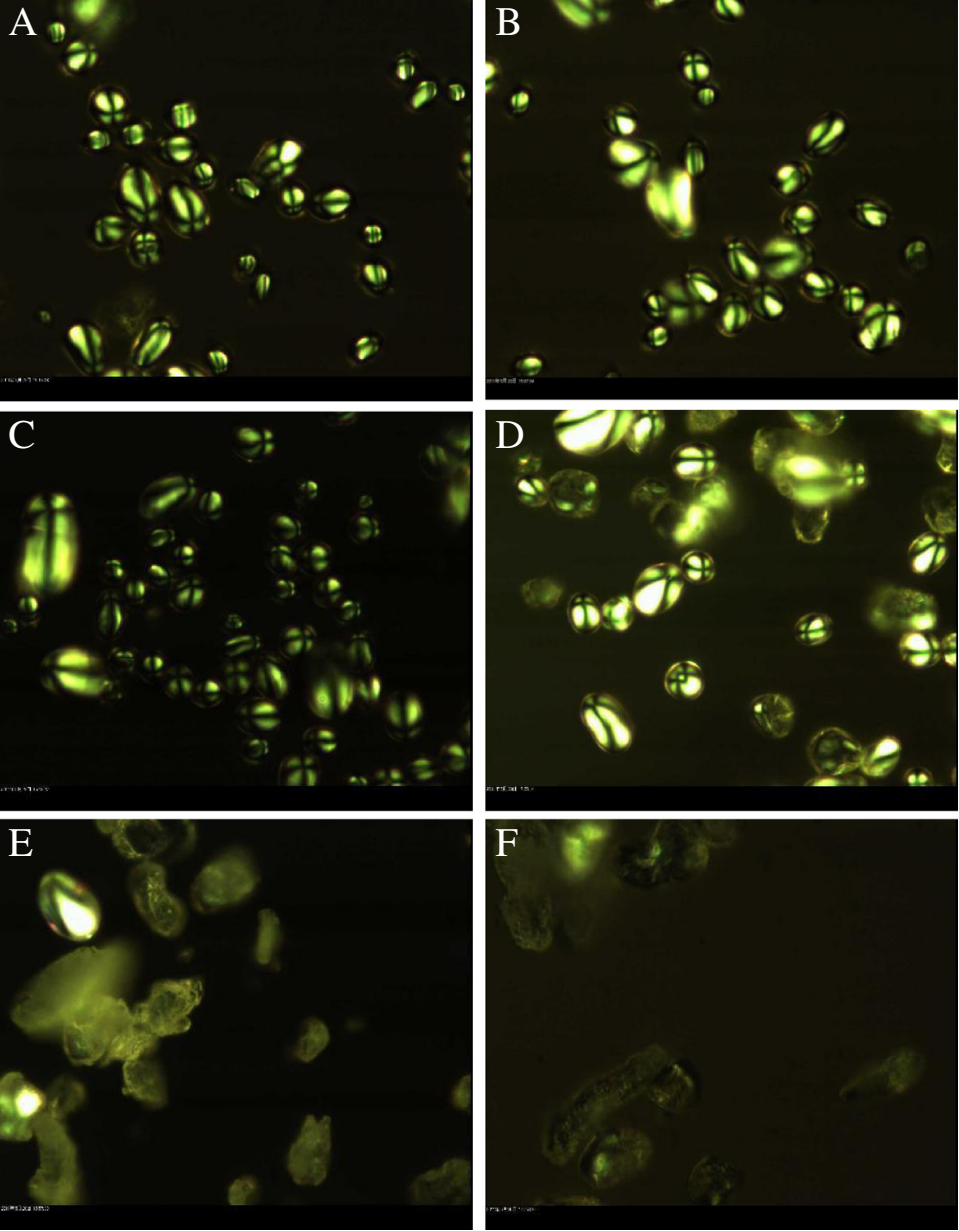
**PLM micrographs of native and microwave-treated starches. A**, **B**-Native starch: **C**, **D**, **E**, **F**-microwave-treated starch for 5,10,15 and 20 s respectively.

The crystalline structure of microwave-treated potato starch granules was observed by PLM [15]. The micrographs of native and microwave-treated starch granules are shown in Figures 2A, B where the native starch granules each present a distinct, characteristic Maltese cross. The amylose and amylopectin chains are arranged radially within the granule and the density and refractive index difference in crystalline and amorphous structure show the anisotropy and generates birefringence when observed with a microscope under polarised light [15-17]. After microwave treating for 5 s, the Maltese cross of starch granules displayed slight changes, which suggest that the radial arrangement of the chain axis of the starch granules was very lightly influenced by a short time of microwave processing (Figure 2C). Half of starch granules lost birefringence after microwave treating for 10 s (Figure 2D), which means that the microwave energy vibrates the water molecules present in the crystalline regions of the starch granules thereby destroying the lamellar arrangement of the amylopectin crystals even below gelatinization temperature [3]. The birefringence of granules disappeared entirely after 15 and 20 s (Figure 2E-F), which was likely that the vibrational motion of the polar water molecules during microwave heating directly impact the crystalline lamella of the starch chain and destroy the lamellar arrangement of crystalline region [18]. The loss of crystallinity was progressive under increasing duration of microwaving, which was consistent with the destroyed surfaces observed under SEM.

### Molecular weight distribution (MWD) by GPC-MALS

**Figure 3:**
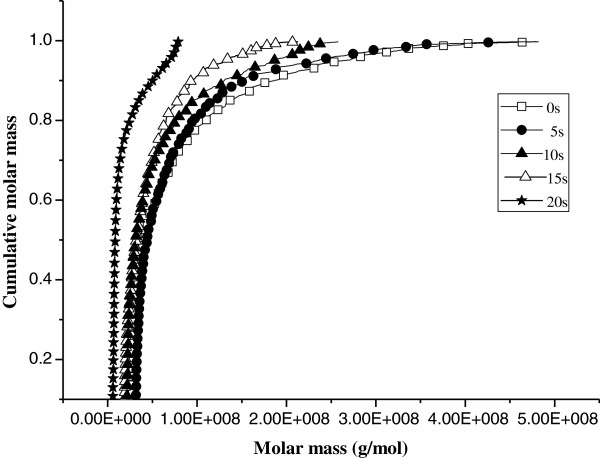
**Cumulative molar mass of molecular weight distribution of native and microwave-treated starches.** □-Native starch; ●-microwave-treated starch for 5 s; ▲-microwave-treated starch for 10s; ▽-microwave-treated starch for15 s; ★-microwave-treated starch for 20 s.

The cumulative molar mass of molecular weight distribution of potato starch subjected to a microwave intensity of 2450 MHz and 750 W as a function of time as determined by GPC is shown in Figure 3. With increasing the microwave treating time from 0 to 20s, the cumulative molar mass curves shifted to lower molecular weight. The molecular weight slowly changed over 5 ~ 15 s but changed abruptly at the time of 20s. For example, the molecular weight (Mw) of Native potato starch of 7.62 × 10^7^ Da was reduced to 7.25 × 10^7^, 5.42 × 10^7^, 4.59 × 10^7^ and 1.72 × 10^7^ after 5, 10, 15 and 20s of microwave treatment, respectively. Starch with high molecular weight above 2 × 10^8^ Da was so sensitive to microwave treatment, so the relatively high molecular weight fragments were gradually fractured into the low molecular weight fragments as the microwave time prolonged, which is likely the result of the strong vibrational motion of the polar molecules due to the application microwave energy [18]. It is noteworthy that the percentage of above 2 × 10^8^ Da fragment decreased from 8.4%,6.4%,4.0%,0.4% to 0; meanwhile, the percentage of under 5 × 10^7^ Da fragment increased from 57.2%,58.0%,68.6%,71.1% to 90.0% after 0, 5, 10,15 and 20s of microwave treatment, respectively. This result agreed with reported literatures using microwave treatment for starch degradation [19,20].

### Effect of microwave treating on apparent viscosity

**Figure 4:**
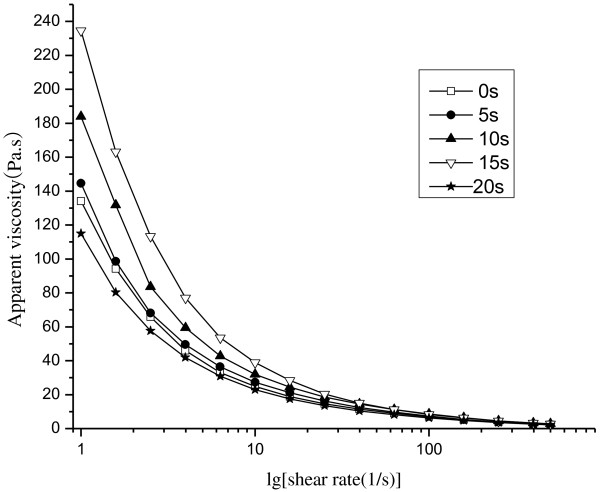
**Apparent viscosity vs. shear rate of native and microwave-treated starches.** □-Native starch; ●-microwave-treated starch for 5 s; ▲-microwave-treated starch for 10s; ▽-microwave-treated starch for15 s; ★-microwave-treated starch for 20 s.

The effect of microwave treating on the apparent viscosity of starch pastes at a specified shear rate is shown in Figure 4. The apparent viscosity decreased as shear rate increased, which indicates that native and microwave-treated potato starch classify as non-Newtonian fluids and present shear-thinning behavior. Shear thinning is related to the progressive orientation of molecules in the direction of flow and breaking of H-bonds formed in the amylose-amylopectin-water structure during shearing [21]. The magnitudes of apparent viscosity obtained at each shear rate increased with increasing time of microwave treating from 0 to 15 s; nevertheless viscosity decreased with continued microwave treating from 15 to 20 s. The increase in apparent viscosity was generally ascribed to the granules was ruptured and gelatinized gradually as function of the vibrational motion of the polar molecules during microwave treating [2,18]. The decrease in apparent viscosity can be explained by degradation of starch molecules under longer microwave treating from 15 to 20 s. Higher reaction temperatures and longer treating times promoted degradation of starches (i.e. wheat, barley, potato, rice, corn and waxy corn) [14,22].

### Effect of microwave treating on dynamic rheological properties of potato starch

**Figure 5:**
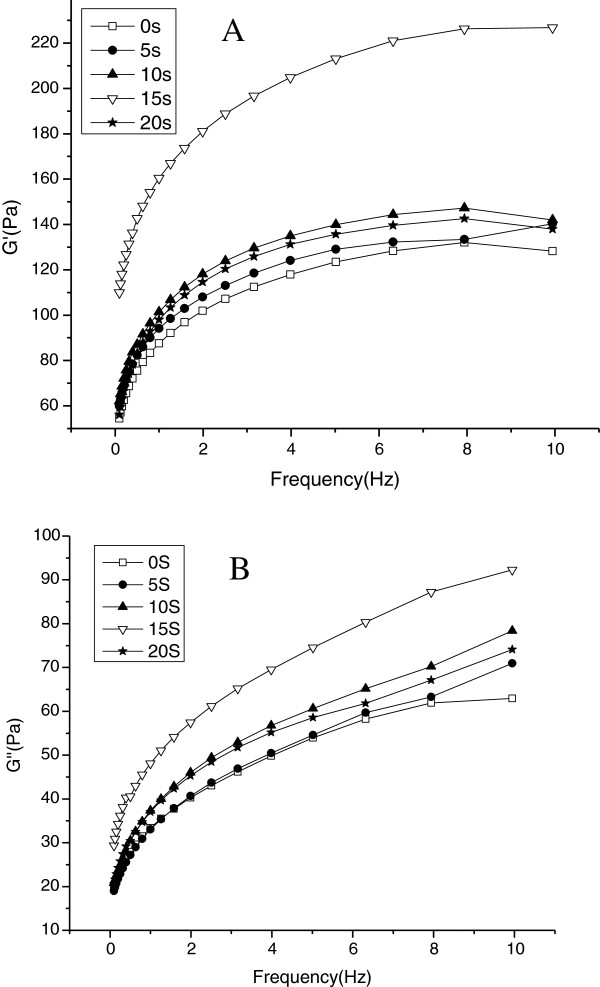
**Plot of *G’ *and log *G” *versus frequency of native and microwave-treated starches. A-*G’ *B-*G”*** □-Native starch; ●-microwave-treated starch for 5s; ▲-microwave-treated starch for 10s;▽-microwave- treated starch for 15s; ★-microwave- treated starch for 20s.

The viscoelastic behavior of starch pastes was measured using the dynamic mechanical storage modulus (*G*’) and loss modulus (*G”*), which reflect elasticity and viscosity, respectively [23]. In Figure 5, *G’* and *G”* increased over the frequency range for the test samples, where *G’* was higher than *G”* indicated that elasticity prevailed over viscosity [21]. The magnitudes of *G’* and *G”* increased as microwave treating time progressed from 0 to 15 s but both decreased with continued treating from 15 to 20 s. The ordered structure of starch granules is disrupted when native starch is treated by microwave, which is a series of events that occur progressively until the granules are completely disrupted from 0 to 15 s [3]. The increases in *G’* and *G”* depend on the extent of gelatinization and the development of a network relating to those chains leached from the starch granules during the microwave treatment [24]. On the contrary, when the relatively high molecular weight fragments were fractured into low molecular weight as the microwave time prolonged from 15 to 20s with the strong vibrational motion of the polar molecules, the longer chain did not appear to be contributing to the continuous network needed for gel formation, so the storage modulus *(G’)* and loss modulus *(G”)* decreased [18]. The experimental data of SEM, PLM, molecular weight distribution and rheological properties during microwave treating of potato starch showed a good support each other.

### Experimental

#### Potato starch extraction

Starch extraction was carried out as described by Singh et al. [25]. Fresh intact potatoes were chosen, washed, peeled, diced, dipped in distilled water containing a small amount of potassium metabisulfite, and finally milled on a cutter (DS-1 high speed tissue stamp mill, Shanghai Specimen Model Factory, China) was washed with distilled water. Filtrate was collected in a glass beaker and kept undisturbed overnight. The supernatant was then decanted off the solid layer of starch. This was repeated 4 to 5 times until the supernatant became transparent. The starch cake was collected and naturally air-dried at room temperature.

#### Preparation of microwave-treated samples

Dispersion of potato starch in distilled was prepared to get final solids concentration of 33% (w/w) on a dry weight basis and stirred for 2 min in order to ensure full suspension. The starch dispersions were covered and then treated in a microwave oven (Galanz) at 2450 MHz and 700 W for 0, 5, 10, 15 and 20 s and sample temperatures were measured by using a T-type thermocouple immediately following the heating treatment. The recorded temperatures are the average sample temperatures. The position of the sample in the microwave was always placed at the same place within the oven to avoid any change in power absorbed. Multiple preliminary tests were conducted to select the conditions required for each treatment [3].

#### Scanning electron microscopy (SEM)

The native and microwave-treated starch granules were studied by SEM (Quanta-200, FEI Company, Netherlands). All samples were mounted on aluminum stubs using double-sided cellophane tape and coated with a thin film of gold (10 nm) before examination at an accelerating voltage of 5 kV [26].

#### Polarized light microscopy analysis (PLM)

A polarization microscope (Leica DM2500P, Leica Microsystems, Inc., Wetzlar, Germany) was used at 500× magnification. The granules were dispersed in glass vials at 10 mg starch in 1 mL of distilled water. One drop of starch suspension was then transferred onto a slide under a slip cover. Each sample was photographed under polarized light [27].

#### Gel permeation chromatography (GPC) coupled with multiangle light scattering (MALS)

Gel permeation chromatography (GPC) (Waters, America) coupled with MALS (Wyatt, America) was performed to determine the molecular weight. Each starch sample (5 mg) was mixed with Dimethyl Sulphoxide (DMSO )(10 mL) containing 0.05 mM LiBr at 60°C for 12 h and then filtered through 5 mm membrane filter (Millipore Co.,USA). The mobile phase was DMSO at a flow rate of 0.5 mL/min and detection was achieved with refractive index detector. A chromatographic column (Styragel HMW 6E, Waters, America) and the column oven were maintained at 25°C. The wavelength of 658 nm laser was used and the data of light scattering was collected and analyzed [28].

#### Flow and dynamic rheological measurements of potato starch

The steady and dynamic shear properties of the potato starch were obtained using a rheometer (AR 1000, TA Instruments, New Castle DE, USA) with a parallel plate system (40 mm ∅) at a gap of 1 mm. Each sample of concentration 33% (w/w) was transferred to the rheometer plate at 25°C and excess material was wiped off with a spatula. The exposed edges of the samples were covered with silicon oil and cover plates to preclude drying during measurement. Steady shear data were obtained for shear rates across 0 to 500 s^-1^. Dynamic shear data were obtained from shear frequency sweeps over 0 to 10 Hz; storage modulus (*G’*) and loss modulus (*G”*) values were recorded. All rheological measurements were performed in triplicate [25].

## Conclusions

The results of this study show that microwave treating affects the morphology, crystalline structure, molecular weight distribution and rheological properties of potato starch granules. According to investigation under SEM and PLM, microwave treating induced marked changes in the structure of potato starch granule morphology. Native starch granules showed a clear and regular elliptical shape with smooth surfaces. Flaws and fractures appeared when granules were subjected to 5 to 10 s microwave treating and most were deformed until rupture after treating was prolonged to 15 to 20 s. Native starch granules generated birefringence, as evidenced from clear characteristic Maltese crosses visible under PLM. The birefringence gradually decreased and even disappeared with microwaving from 5 to 20 s, which can be explained by damage to the orderly arrangement of crystalline regions during microwave treating. The molecular weight (Mw) values of potato starch and the proportion of large M_W_ fraction were considerably reduced with increasing the microwave treating time from 0 to 20 s. The susceptibility of long molecular chain above 2 × 10^8^ Da is so high that those were gradually fractured into the low and moderate molecular weight fragments as the microwave time prolonged. From rheometer readings, the steady and dynamic rheological properties of potato starch paste show that apparent viscosity decreased with increasing shear rate and presented shear-thinning behavior. The dynamic rheological data for storage (*G*’) and loss (*G”*) moduli as a function of frequency showed that *G*’ and *G”* increase when subjected to microwave treating from 0 to 15 s, which is attributed to the intermolecular association of the amylose and amylopectin chains leached by microwave energy from the granules during the fracture of potato starch crystal structure. *G’* and *G”* decreased as microwave treating continued from 15 to 20 s, which was correlated with the degradation of dextran chains during further microwave treating.

## Competing interests

The authors declare that they have no competing interests.

## Authors’ contributions

YLX made a significant contribution to experimental design, data analysis and manuscript preparation. MXY participated in the experiments. SSY participated in the experiments and made a substantial contribution to experimental design and data analysis. SMS participated in method development and validation. QGH participated in data analysis of polarized light microscopy analysis (PLM). All authors read and approved the final version of the manuscript.
